# A meta-analysis of the efficacy of programmed cell death 1/its ligand inhibitors plus cytotoxic T-lymphocyte-associated antigen 4 inhibitors in non-small cell lung cancer

**DOI:** 10.3389/fphar.2024.1267763

**Published:** 2024-02-05

**Authors:** Li Lin, Lu Xiao, Lei Li, Chen Chen, Haorong Zhang, Changyan Yu, Lanfang Zhang, Anhua Wei, Wei Li

**Affiliations:** ^1^ Department of Oncology, Wuhan Asia General Hospital, Wuhan, China; ^2^ Department of Rehabilitation, Tongji Hospital, Tongji Medical College, Huazhong University of Science and Technology, Wuhan, China; ^3^ Department of Pharmacy, Tongji Hospital, Tongji Medical College, Huazhong University of Science and Technology, Wuhan, China

**Keywords:** CTLA-4 inhibitor, PD-1/PD-L1 inhibitor, non-small cell lung cancer, meta-analysis, NSCLC

## Abstract

**Background:** Immune checkpoint inhibitors (ICIs), either as monotherapy or in combination with chemotherapy, have improved the therapeutic outcome for non-small cell lung cancer (NSCLC). However, the efficacy of combination therapies, such as programmed cell death 1(PD-1)/its ligand (PD-L1) and cytotoxic T-lymphocyte-associated antigen 4 (CTLA-4) inhibitors, in targeting different pathways remains unclear. We performed a meta-analysis to determine whether the addition of a CTLA-4 inhibitor to PD-1/PD-L1 therapy improves the efficacy of PD-1/PD-L1 monotherapy in NSCLC.

**Methods:** We systematically searched various electronic databases for suitable trials. Only randomized controlled trials (RCTs) comparing the clinical efficacy of PD-1/PD-L1 with and without CTLA-4 were included in the analyses. The meta-analysis software RevMan 5.3 was used for statistical analyses.

**Results:** A total of seven RCTs were retrieved. The results suggested that the combination of CTLA-4 and PD-1/PDL-1 inhibitors did not show enhanced efficacy over PD1/PDL-1 inhibitor monotherapy as determined by overall survival (OS) (HR = 0.98, 95% CI = 0.84–1.14, *p* = 0.79), progression-free survival (PFS) (HR = 0.92, 95% CI = 0.81–1.06, *p* = 0.25), and objective response rate (ORR) (HR = 1.08, 95% CI = 0.96–1.21, *p* = 0.19). Furthermore, the combination immunotherapy was associated increased toxicity as evidenced by increased incidence of any type adverse events (AEs) (RR = 1.06, 95% CI = 1.00–1.13, *p* = 0.03), grade ≥3 immune-mediated AEs (RR = 1.58, 95% CI = 1.36–1.82, *p* < 0.05), and treatment discontinuation (RR = 1.83, 95% CI = 1.46–2.28, *p* < 0.05).

**Conclusion:** Combining anti-CTLA-4 with anti-PD-1/PD-L1 therapy did not improve the therapeutic efficacy, and was associated with greater toxicity than anti-PD-1/PD-L1 monotherapy in patients with advanced NSCLC. Further investigation of the combination immunotherapy in specific subsets of patients is warranted to identify and define the patient-specific benefits of this combination.

**Systematic Review Registration:** https://www.crd.york.ac.uk/prospero/, identifier CRD42023435399

## Background

In recent years, monoclonal antibodies (mAbs) have revolutionized cancer therapy. Immunotherapy with monoclonal antibodies targeting programmed cell death 1 (PD-1) or its ligand (PD-L1) have become the standard salvage therapy approved for the treatment of advanced non-small cell lung cancer (NSCLC), either as a monotherapy or in combination with chemotherapy (Gandhi et al., 2018; Paz-Ares et al., 2018; Socinski et al., 2018).

Because of the limitations of treatment-related toxicities and PD-L1 tumor proportion score, only a minority of patients demonstrate notable anti-tumor effects (Camidge et al., 2019), and the efficacy of ICI combinations over that of PD-1/PD-L1 monotherapy remains under detable. Some meta-analyses had been done previously. A meta-analysis by Shen et al.reported that the PD-1/PD-L1suppressors in combination with conventional chemotherapy have promising ORR rate and survival efficacy (Shen et al., 2023). Another recent research by Chen et al.demonstrated that PD-1/PD-L1 inhibitors plus anti-angiogenic agents obviously enhance the efficacy and safety as second or later-line therapy in NSCLC (Chen et al., 2023).

Monoclonal immunoglobulin G2 antibodies targeting cytotoxic T-lymphocyte-associated antigen 4 (CTLA-4) prevent normal downregulation of T cells and prolong T-cell action, thereby enhancing immune function (Tarhini and Kirkwood, 2008). Previous studies have shown an additive or synergistic antitumor activity of simultaneous blockade of the PD-1/PD-L1 and CTLA-4 pathways, and support the combination as a therapeutic option for patients with low/negative PD-L1 expression (Antonia et al., 2016; Hellmann et al., 2017; Hellmann et al., 2018).

However, there are still conflicting reports on the benefits of the combination therapies because of toxicity, lack of therapeutic efficacy, or because of differences in response arising from variations in tumor mutational burden (TMB) and PD-1 expression levels (Liu et al., 2021). Therefore, more evidence is needed to demonstrate that the addition of CTLA-4 to PD-1/PD-L1 therapy is superior to PD-1/PD-L1 monotherapy in NSCLC.

We conducted a meta-analysis to determine whether the addition of a CTLA-4 inhibitor to PD-1/PD-L1 therapy improves the efficacy of PD-1/PD-L1 alone in NSCLC.

## Materials and methods

### Search strategy

A literature search of studies published until June 2023 in the PubMed, Embase, and Cochrane databases was performed by two independent reviewers. The keywords and relevant Medical Subject Heading (MeSH) terms used for the searches included the following: “Pembrolizumab,” “Nivolumab,” “Atezolizumab,” “Cemiplimab,” “Avelumab,” “Durvalumab” and “Ipilimumab,” “Tremelimumab” and “Non-small cell lung cancer.” Reference lists and materials were manually retrieved to identify potentially eligible articles.

### Eligibility criteria

Inclusion criteria were as follows: (Gandhi et al., 2018): participants: studies that enrolled patients diagnosed with NSCLC; (Socinski et al., 2018); interventions: comparing the clinical efficacy of PD-1/PD-L1 with or without CTLA-4; (Paz-Ares et al., 2018); outcomes: overall survival (OS), progression-free survival (PFS), objective response rate (ORR), and adverse events (AEs); and (Camidge et al., 2019) study design: randomized controlled trials (RCTs).

### Quality assessment

All the cohort articles were assessed for risk of bias using the Cochrane Collaboration’s “risk of bias” tool for the RCTs (Higgins et al., 2011). The process was conducted in two separate studies, and disagreements were resolved by discussion.

### Data extraction

Two reviewers independently extracted the following information: author’s name, year of publication, trial, therapy arm, follow-up period, number of patients, mean patient age, and relevant outcome data. Disagreements were resolved through discussion. Publication bias was evaluated using funnel plots.

### Data synthesis and analysis

The experimental group was defined as the one receiving the combination immunotherapy and the control group as that receiving anti-PD-1/PD-L1 monotherapy. Heterogeneity of the articles were assessed using the *I* ^2^ statistic and Chi-square test (Higgins and Thompson, 2002). *I* ^2^ ≥ 50% was considered to indicate high heterogeneity, whereas *I* ^2^ < 50% was suggested to indicate low heterogeneity (Higgins et al., 2003). The fixed-effects model was used when there was a low degree of heterogeneity among the studies; otherwise, the random-effects model was used. Statistical significance was set at *p* < 0.05. Review Manager version 5.3 software (RevMan; The Cochrane Collaboration, Oxford, United Kingdom) was used for statistical analysis. The results are shown as forest plots.

## Results

### Study selection

**FIGURE 1:**
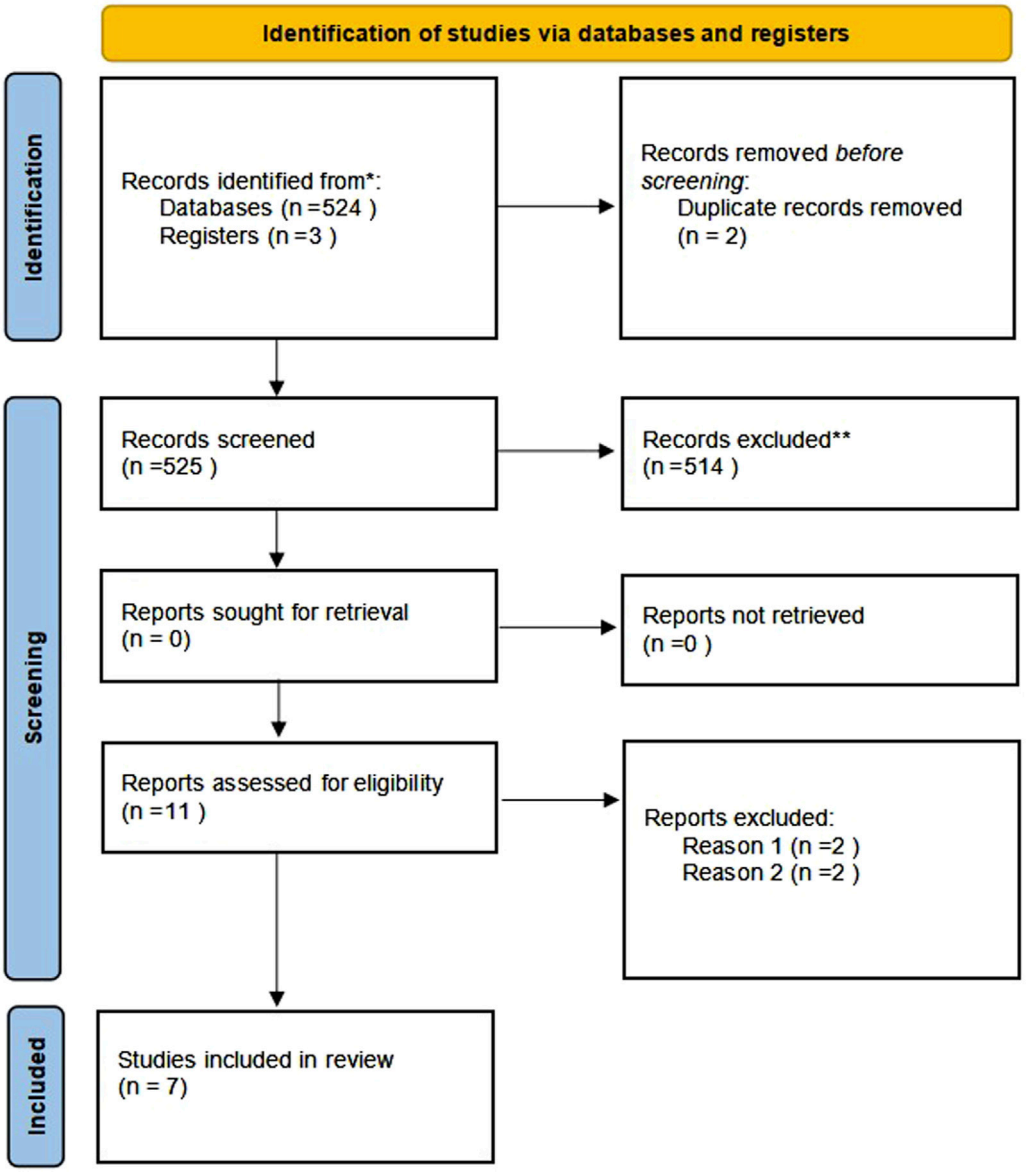
PRISMA flow chart of selection process to identify studies eligible for pooling Reason 1.

**FIGURE 2:**
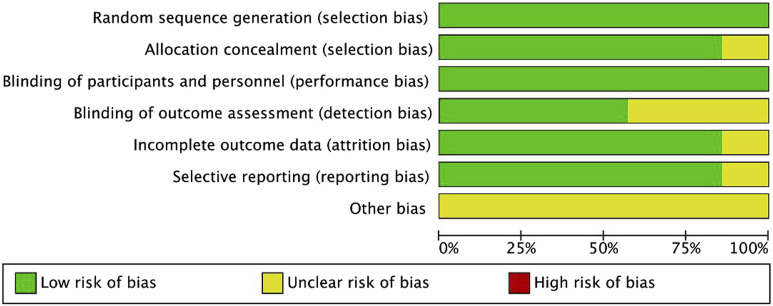
Methodological quality assessment for each included study.

**FIGURE 3:**
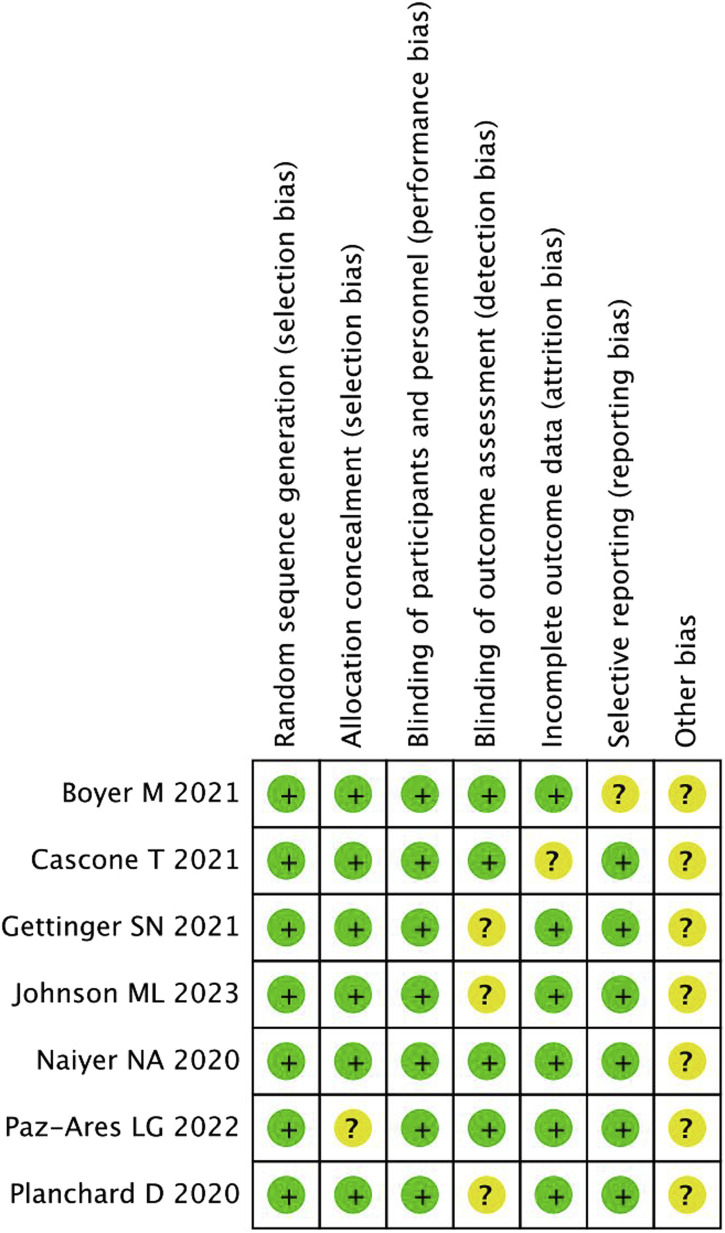
Quality assessment summary for included studies.

A total of 527 publications were retrieved. Following a review of the titles and abstracts, 11 studies were evaluated by reading the complete article. However, four of these were excluded based on the inclusion criteria. Finally, seven RCTs were included in the analyses (Planchard et al., 2020a; Rizvi et al., 2020; Boyer et al., 2021; Cascone et al., 2021; Gettinger et al., 2021; Paz-Ares et al., 2022; Johnson et al., 2023). Figure 1 illustrates the search process in detail. Figures 2, 3 summarize the quality assessment process.

All included publications were based on moderate-quality evidence. Table 1 describes the primary characteristics of the eligible studies in detail.

**TABLE 1:** Characteristics of the include studies.

Author	Boyer M		Planchard D		Naiyer NA		Gettinger SN		Paz-Ares LG		Cascone T		Johnson ML	
Year	2021		2020		2020		2021		2022		2021		2023	
trial	NCT03302234		NCT02352948		NCT02453282		NCT02785952		NCT02477826		NCT03158129		NCT03164616	
follow-up period	2018.1.12–2019.8.12		2015.1.13–2016.9.13		2015.7.21–2018.10.30		2015.12–2018.4		2015.8.5–2016.11.30		2017.6–2018.11		—	
therapeutic regimen	P + I	P	D + T	D	D + T	D	N + I	N	N + I	N	N + I	N	D + T + CT	D + CT
	P:200 mg, q3w + I:1 mg/kg, q6w	P:200 mg, q3w + placebo	D: 20 mg/kg q4w + T: 1 mg/kg q4w	D:10 mg/kg q2w	D: 20 mg/kg q4w + T: 1 mg/kg q4w	D:20 mg/kg q4w	N:3 mg/kg q2w + I:1 mg/kg q6w	N:3 mg/kg q2w	N:3 mg/kg q2w + I:1 mg/kg q6w	N:240 mg q2w	N:3 mg/kg q2w + I:1 mg/kg q6w	N:3 mg/kg q2w	T; 75 mg D:1500 mg q3w 4curcles + D:1500 mg q4w	D:1500 mg q3w 4curcles + D:1500 mg q4w
Patients	284	284	174	117	372	374	138	137	396	396	21	23	338	338
Median Age	64	65	62.5	63	65	64	67.5	68.1	64	64	65	66.1	63	64.5
Outcomes	OS:HR:1.08 (0.85–1.37)		OS:HR:0.98 (0.74–1.30)		AEs:RR:1.12 (0.99–1.27)		OS:HR:0.87 (0.66–1.15)		OS:HR:0.19 (0.01–4.58)		ORR:RR:0.88 (0.27–2.83)		ORR:RR:0.93 (0.74–1.18)	
	PFS:HR:1.06 (0.86–1.31)		PFS:HR:0.87 (0.68–1.11)		—	—	PFS:HR:0.80 (0.61–1.05)		PFS:HR:0.52 (0.11–2.46)		—	—	AEs:RR:1.05 (1.00–1.10)	
	ORR:RR:1.00 (0.84–1.20)		ORR:RR:0.97 (0.56–1.69)		—	—	ORR:RR:1.08 (0.65–1.81)		ORR:RR:1.32 (1.07–1.62)		—	—	—	—
	AEs:RR:1.03 (0.99–1.08)		AEs:RR:0.99 (0.92–1.05)		—	—	AEs:RR:1.12 (0.91–1.37)		AEs:RR:1.17 (1.07–1.27)		—	—	—	—

P, pembrolizumab; I, ipilimumab; D, durvalumab; T, tremelimumab; N, nivolumab; CT, Chemotherapy; HR, hazard ratio; RR, risk ratio.

### Clinical and methodological heterogeneity

#### OS

**FIGURE 4:**
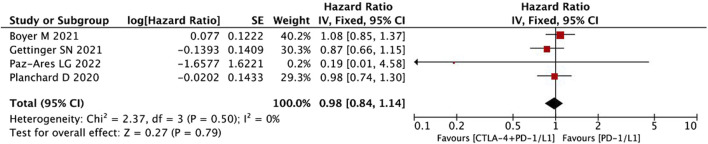
Pooled analysis of OS.

Because there was no heterogeneity among the studies, we applied a fixed-effects model to the relevant analysis. The pooled result for OS showed no significant benefit of the combination immunotherapy over anti-PD-1/PD-L1 monotherapy (HR = 0.98, 95% CI = 0.84–1.14, *p* = 0.79) (Figure 4).

#### PFS

**FIGURE 5:**
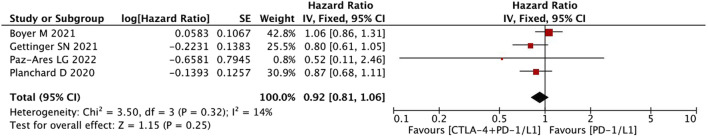
Pooled analysis of PFS.

A fixed-effects model was used to analyze the pooled PFS data because heterogeneity across the included studies was low. The pooled data for PFS did not show any significant effect of the combination immunotherapy over anti-PD-1/PD-L1 monotherapy (HR = 0.92, 95% CI = 0.81–1.06, *p* = 0.25) (Figure 5).

#### ORR

**FIGURE 6:**
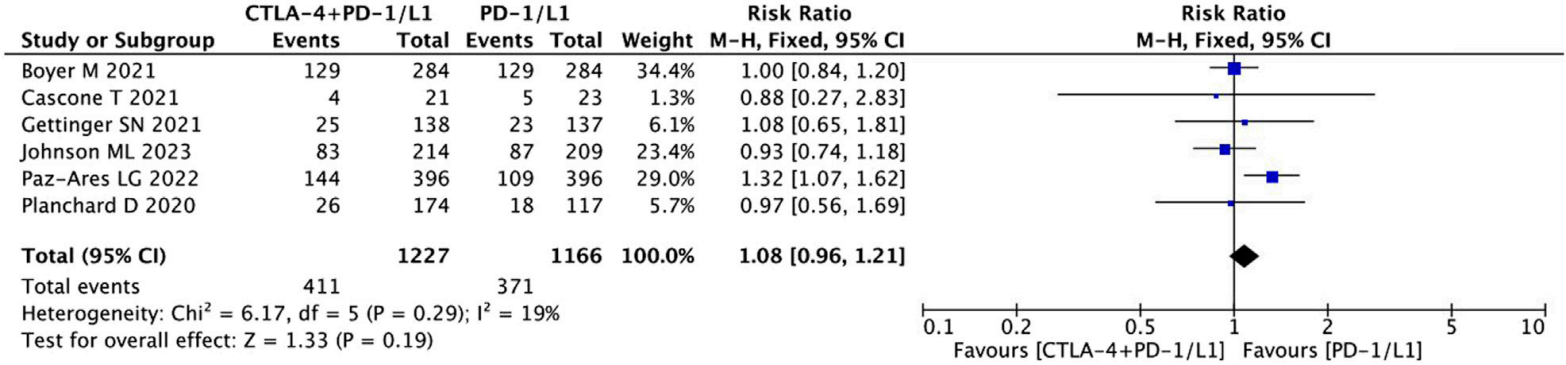
Pooled analysis of objective response rates (ORR).

The ORR showed no significant difference between the two treatment regimens in the fixed-effects model (HR = 1.08, 95% CI = 0.96–1.21, *p* = 0.19) (Figure 6). The addition of CTLA-4 to PD-1/PD-L1 therapy did not improve the ORR compared to PD-1/PD-L1 alone in NSCLC.

#### AE

**FIGURE 7:**
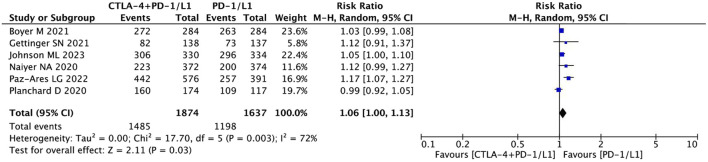
Pooled analysis of adverse effects (AEs).

**FIGURE 8:**
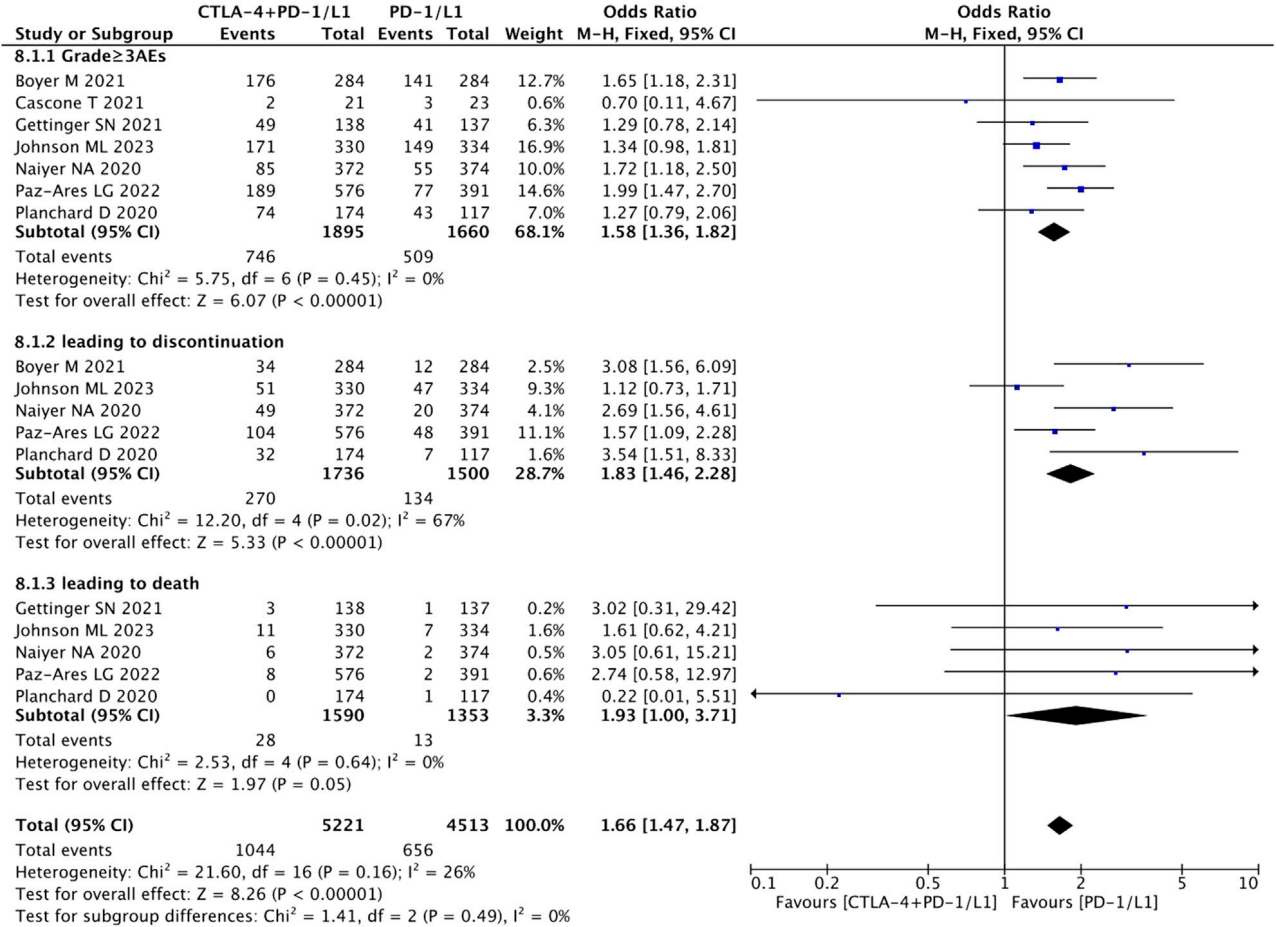
Pooled analysis of sub-group adverse effects.

The combination immunotherapy arm was associated with increased rates of any type AEs (RR = 1.06, 95% CI = 1.00–1.13, *p* = 0.03) (Figure 7), higher grade 3 AEs (RR = 1.58, 95% CI = 1.36–1.82, *p* < 0.05), and AEs leading to treatment discontinuation (RR = 1.83, 95% CI = 1.46–2.28, *p* < 0.05) compared with the antiPD-1/PD-L1 monotherapy arm. Whereas, the pooled data showed that the rate of AEs leading to death (RR = 1.93, 95% CI = 1.00–3.71, *p* = 0.05) was not significantly different between the two treatment regimens (Figure 8).

#### Publication Bias

**FIGURE 9:**
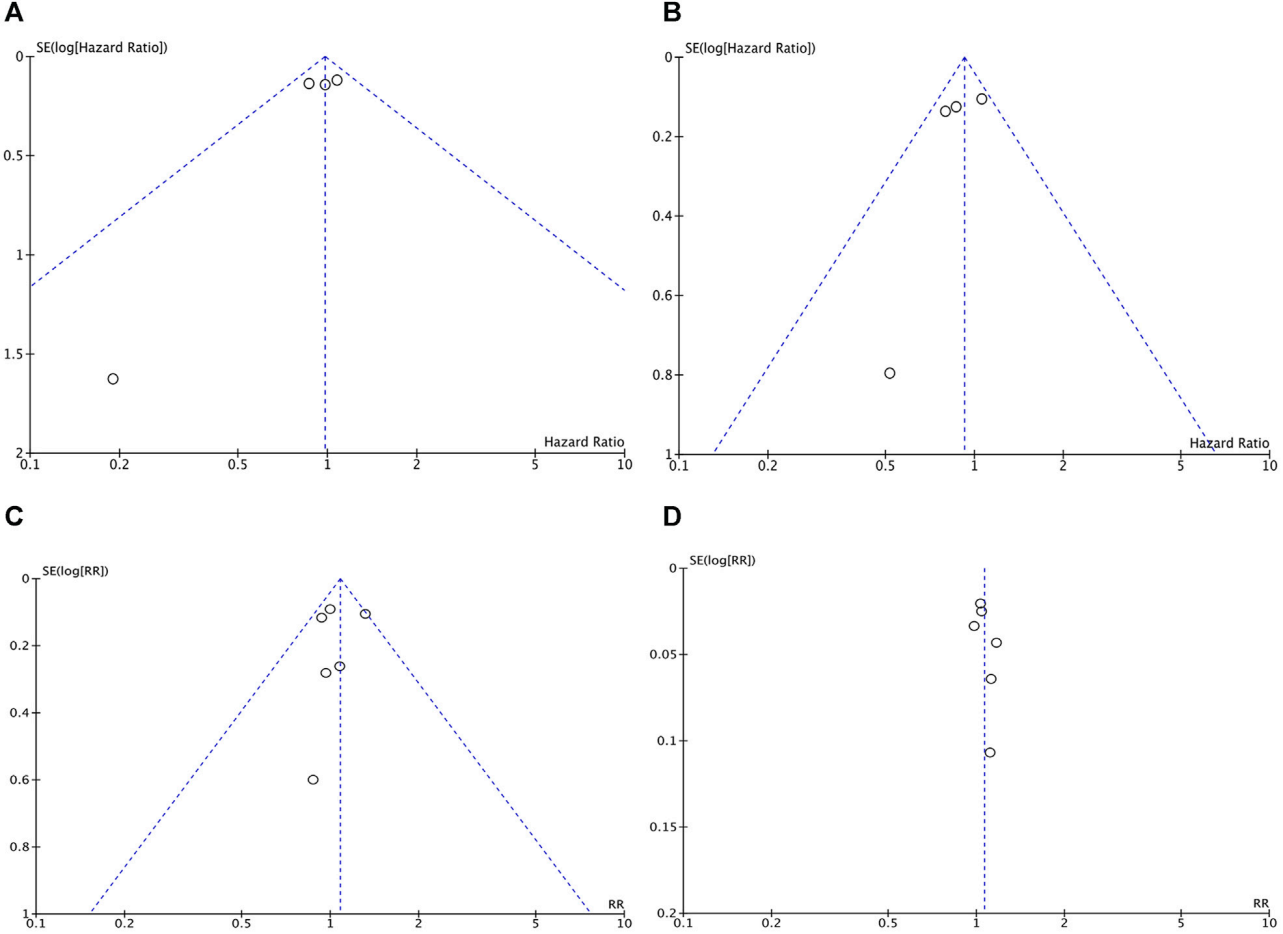
The funnel plots of the OS **(A)**, PFS **(B)**, ORR **(C)**, and any type of AEs **(D)**.

Forest plots were used to present publication bias. Figure 9 shows funnel plots of the OS (Figure 9A), PFS (Figure 9B), ORR (Figure 9C), and any type of AEs (Figure 9D).

## Discussion

The combination of anti-PD-1/PD-L1 therapy with anti-CTLA-4 is considered to amplify the anti-tumor T-cell responses through non-redundant immune checkpoint blockade, and provide additive or synergistic antitumor activity. However, previous trials suggest that this combination does not provide any additional benefits beyond that of PD-1 inhibition alone (Gettinger et al., 2021).

To determine the efficacy of PD-1/PD-L1, with or without CTLA-4, we performed a comprehensive meta-analysis, and evaluated the benefits and risks of the combination immunotherapy *versus* PD-1/PDL-1 inhibitor monotherapy.

From a biological perspective, the combination immunotherapy should provide superior efficacy compared to PD-1/PDL-1monotherapy. The results of the Lung-MAP S1400I trial (Gettinger et al., 2021) and the CheckMate 227 trial (Paz-Ares et al., 2022) have supported this view. However, we found that adding CTLA-4 inhibitors to PD-1/PD-L1 therapy did not significantly improve the antitumor efficacy indices (survival outcomes including PFS and OS and drug response including ORR) compared to those of PD-1/PD-L1 inhibitors alone.

These findings may be explained by the influence of differences in PD-L1 expression levels and TMB, which have been mentioned in previous trials (Planchard et al., 2020b). PD-L1 expression, measured by immunohistochemistry, is currently the most widely used decision-making tool in clinical practice for selecting patients who will derive the greatest benefit from ICIs, at least in a first-line setting (Reck et al., 2016).

PD-L1 negative tumors do not respond to ICIs. Some reports have indicated a trend toward a better response rate associated with increased PD-L1 expression levels. The analysis of the POSEIDON (Johnson et al., 2023) has demonstrated that patients with PD-L1–low/negative are more likely to show primary resistance to anti–PD-(L)1 therapy. Paz-Ares LG (Paz-Ares et al., 2022) shown that efficacy benefit with nivolumab plus ipilimumab *versus* nivolumab monotherapy for both PD-L1 expression greater than or equal to 1% and 50%. These findings suggest the existence of inherent differences in the immune milieu associated with PD-L1 expression levels, and the complex relationship between tumors and the immune system. However, the optimal cut-off value of PD-L1 expression has not yet been defined.

TMB has recently emerged as a biomarker, independent of PD-L1 expression, for identifying patients who may clinically benefits from ICI therapies (Carbone et al., 2017; Hellmann et al., 2018; Ready et al., 2019). Previous NSCLC trials revealed that PD-1/CTLA-4 combination blockade improved PFS in patients with high TMB, independent of PD-L1 expression (Hellmann et al., 2018; Ready et al., 2019). However, the OS was similar regardless of the TMB level (Jiang et al., 2018). In our opinion, the cut-off point for TMB may provide a reasonable explanation for this observation. In their study, Gettinger et al. (2021) reported that a high TMB (defined as a cut-off of 10 mut/Mb) did not result in a superior outcome with combination therapy. However, Rizvi et al. (2020) reported that a high TMB was associated with a significant favorable contribution of CTLA-4 in combination therapy vs. that of PD-1 monotherapy. They defined high TMB as a cut-off value of 20 mut/Mb. Because the optimal cut-off for TMB differed across the studies included in our analysis, the predictive effect of TMB on survival outcomes could not be established in our study. Therefore, standardization of TMB calculation and reporting as well as a universal threshold for defining high TMB remain challenges that need to be investigated further.

In terms of AEs, we consistently found that the combination therapy increased the incidence of grade 3 AEs and AEs that lead to discontinuation. This finding indicates that the AEs associated with the combination therapy worsened in patients on treatment, thereby providing minimal benefits from the drugs. We expect that the risk of immune-related AEs can be reduced by carefully selecting patients for treatment with ICI combinations.

The limitations of our study include its retrospective nature and the various ICIs used in the studies included in our analysis, resulting in an imbalance between the two groups. Analyses of subgroups based on the ICIs are warranted to answer these questions. In addition, we could not draw any conclusions regarding the influence of PD-L1 expression level and TMB on ICIs because of the limited data on the covariates available for analysis. Further high-quality studies with additional data are required to clarify this issue.

## Conclusion

In summary, our analysis suggests that addition of CTLA-4 to PD-1 therapy failed to improve the survival efficacy, but also increased the incidence of grade 3 AEs and AEs leading to discontinuation when compared with PD-1/PD-L1 monotherapy. The predictive values of TMB and PD-L1 expression need to be addressed in future studies.

## Data Availability

The original contributions presented in the study are included in the article/supplementary material, further inquiries can be directed to the corresponding authors.

## Abbreviations

NSCLC, Non-small cell lung cancer; PD-1, Programmed cell death 1.
